# Temporal Dynamics of Uncertainty Cause Anxiety and Avoidance

**DOI:** 10.5334/cpsy.105

**Published:** 2024-06-20

**Authors:** Dan Holley, Erica A. Varga, Erie D. Boorman, Andrew S. Fox

**Affiliations:** 1 Department of Psychology, University of California, Davis, Davis CA, 95616, USA; 2 California National Primate Research Center, University of California, Davis, Davis CA, 95616, USA; 3 Center for Neuroscience, University of California, Davis, Davis CA, 95618, USA; 4 Center for Mind and Brain, University of California, Davis, Davis CA, 95616, USA

**Keywords:** fear, anxiety, uncertainty, threat processing, computational modeling

## Abstract

Alfred Hitchcock, film director and “Master of Suspense,” observed that terror is not driven by a negative event, but “only in the anticipation of it.” This observation is not restricted to the movies: Anxiety builds when we anticipate uncertain negative events, and heightened reactivity during uncertain threat anticipation is a transdiagnostic marker of anxiety (Grupe & Nitschke, 2013; Holley & Fox, 2022; Hur et al., 2020; Krain et al., 2008; Simmons et al., 2008; Yassa et al., 2012). Here, we manipulate the temporal dynamics of an uncertain threat to demonstrate how the evolving expectation of threat can lead people to forgo rewards and experience fear/anxiety. Specifically, we show that increased “hazard rate,” which can build during periods of uncertainty, promotes a tendency to avoid threatening contexts while increasing fear/anxiety. These results provide insight into *why* the anticipation of temporally uncertain threats elicits fear/anxiety, and reframe the underlying causes of related psychopathology.

## Introduction

Extreme anxiety and anxiety disorders can impair our ability to function (Fox & Kalin, 2014; Meacham & T. Bergstrom, 2016; Shackman & Fox, 2016). This is not a rare occurrence; anxiety disorders affect roughly 1 in 3 people during their lifetime and are a leading cause of disability worldwide (Bandelow & Michaelis, 2015; Kessler et al., 2012). For many patients suffering from anxiety disorders, anticipatory fear/anxiety while waiting for a negative event is more debilitating than the event itself (Borkovec, 1985; Grupe & Nitschke, 2013). The cumulative effect of anticipatory fear/anxiety can lead people to avoid potentially threatening contexts altogether, thereby missing out on opportunities to thrive.

A major contributor to anticipatory fear/anxiety is “uncertainty,” which is widely accepted as intrinsically anxiogenic (Bandelow & Michaelis, 2015; Borkovec, 1985; Browning et al., 2015; Fox & Kalin, 2014; Grillon et al., 2004) (see Supplemental Text on nomenclature for fear/anxiety). For example, using threat-countdowns, researchers have clearly demonstrated that uncertainty about when a threat will occur heightens anxiety (Hur et al., 2020; Somerville et al., 2013). This has motivated the theories about distinct neural substrates that contribute to uncertain and certain threat processing, but evidence for these theories is mixed (Hur et al., 2020; Somerville et al., 2013; for additional discussion, see Supplemental Text). Reconciling this literature will require understanding *why* temporal uncertainty is anxiogenic. Here, we developed a statistical learning approach to identify parameters that might explain *why* temporal uncertainty elicits fear/anxiety and avoidance behavior. We incorporate this approach in a threat-learning paradigm to disambiguate the effects of probability and hazard rate—which are confounded in previous literature (Davis et al., 2010; Walker et al., 2003; Somerville et al., 2013; Hur et al., 2020)—by holding the probability of threat stable while varying the hazard rate and measuring resulting fear/anxiety.

### Developing a statistical model of uncertain threat anticipation

**Figure 1:**
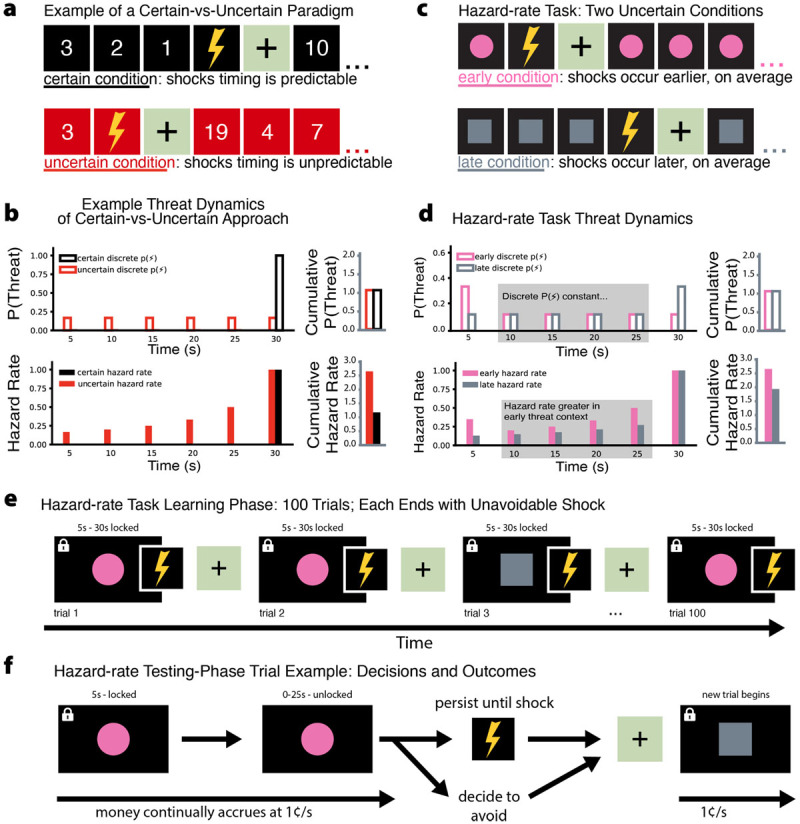
**A computational approach to the study of uncertain threat anticipation. a)** Depiction of threat-anticipation studies to compare certain-vs-uncertain conditions. **b)** P(Threat) (*top*) and hazard rate (*bottom*) reveal that threat-context and hazard rate are confounded, and that the cumulative hazard rate is greater during uncertain trials. **c)** We addressed this confound by comparing two uncertain conditions. **d)** Uncertain conditions differed in the timing of P(Threat) (*top*), but were matched through most of the trial. Cumulatively and during periods of matched P(Threat), the hazard rate (bottom) was higher in the early-threat condition. **e)** Participants were exposed to each condition 50 times in a pseudo-randomized order. All trials ended in shock. **f)** During the testing phase, trials continued and participants earned money until avoidance or shock.

We analyzed past work studying the anxiogenic effects of uncertainty using threat countdowns and modeled the evolving temporal dynamics of threat anticipation during certain and uncertain countdowns. During uncertain threat anticipation we observed a difference between conditions beyond the intended probability manipulation; specifically, we observed increases in the *hazard rate*—that is, an outcome’s probability (P), given that it has not yet occurred (Goel et al., 2010; see Supplemental Text for the formal definition of *hazard rate* and *cumulative hazard rate*). For example, if it is known that a countdown will end with a shock at time 0, the discrete P(Threat) *and* hazard rate values at timepoints 3, 2, 1, and 0 would equal 0, 0, 0, and 1, respectively (Figure 1a, b). In contrast, if there is an equal chance of the shock occurring *at any of those timepoints*, the discrete P(Threat) values would equal .25, .25, .25, and .25, but the hazard rate values would equal .25, .33, .5, and 1. Notably, when the shock is certain to occur at time 0, the cumulative hazard rate is always 1 (e.g., 0 + 0 + 0 + 1 = 1), but in temporally-uncertain threat contexts the cumulative hazard rate is unbounded (e.g., .25 + .33 + .5 + 1 = 2.08). Importantly, this is also true of other widely used tasks that measure fear/anxiety by comparing predictable and unpredictable threats, such as the “NPU-task” (Figure S1; Grillon et al., 2004; Schmitz & Grillon, 2012). While these past paradigms were used to show that temporal uncertainty *is* anxiogenic, our model potentially explains *why* this is the case: probability dynamics evolve as the participant gains information about when the threat *did not* occur, leading to a growing estimate of threat probability. We hypothesized that manipulating the hazard rate would be sufficient to drive avoidance behavior, and that participants’ fear/anxiety ratings would vary with the hazard rate in each trial.

## Methods

### Novel paradigm to manipulate the hazard rate during uncertain threat anticipation

Participants learned and were tested on risk-vs-reward decisions during temporally uncertain threat anticipation trials in two statistically distinct environments: an “early-threat condition” and a “late-threat condition” (Figure 1c, d; see Supplemental Methods for additional details). Critically, in these conditions the middle 20 seconds of each 30-second trial were matched for discrete P(Threat); however, during this 20-second period, the hazard rate of the early-threat condition was *always* higher than that of the late-threat condition (Figure 1d).

Participants (N = 42) learned the statistics of the conditions by receiving 50 unavoidable shocks in each (Figure 1e). No timing information was available to participants. This was followed by a “testing phase” in which participants could persist in the condition to receive incremental cash rewards (i.e. 1¢ per second, for a possible total of $17.50) or end the trial to avoid shocks (Figure 1f). In the testing phase, the delivery of a shock or the decision to “escape”, and thereby avoid the shock, ended the trial. All participants periodically reported their fear/anxiety for a subset of trials in both the learning and testing phases. Following the learning phase, a subset of participants were asked to choose the unique cue, paired with each condition (counterbalanced), that elicited more fear/anxiety. An additional subset experienced a final, 30-second, inescapable-shock trial for each condition (counterbalanced) and rated their fear/anxiety for each. Additional details regarding rating procedures and statistical treatments can be found in Supplemental Methods.

## Results

### Estimated shock-delivery times differ across early- and late-threat environments

After the learning phase, participants self-reported expected shock-delivery times, and reported differences between conditions (N = 42; early mean: 9.48s, SD = 4.32s; late mean: 19.0s (8.70s) and estimated a “tipping point” such that that shocks would be less likely to occur in the early-threat condition compared to the late-threat condition by 16s after trial onset (N = 42; independent-samples *t*-test = 4.87, p < .001; Figure S2). These findings indicated that participants learned the rough threat dynamics of each condition before the testing phase.

### Higher hazard rates cause more avoidance behavior

**Figure 2:**
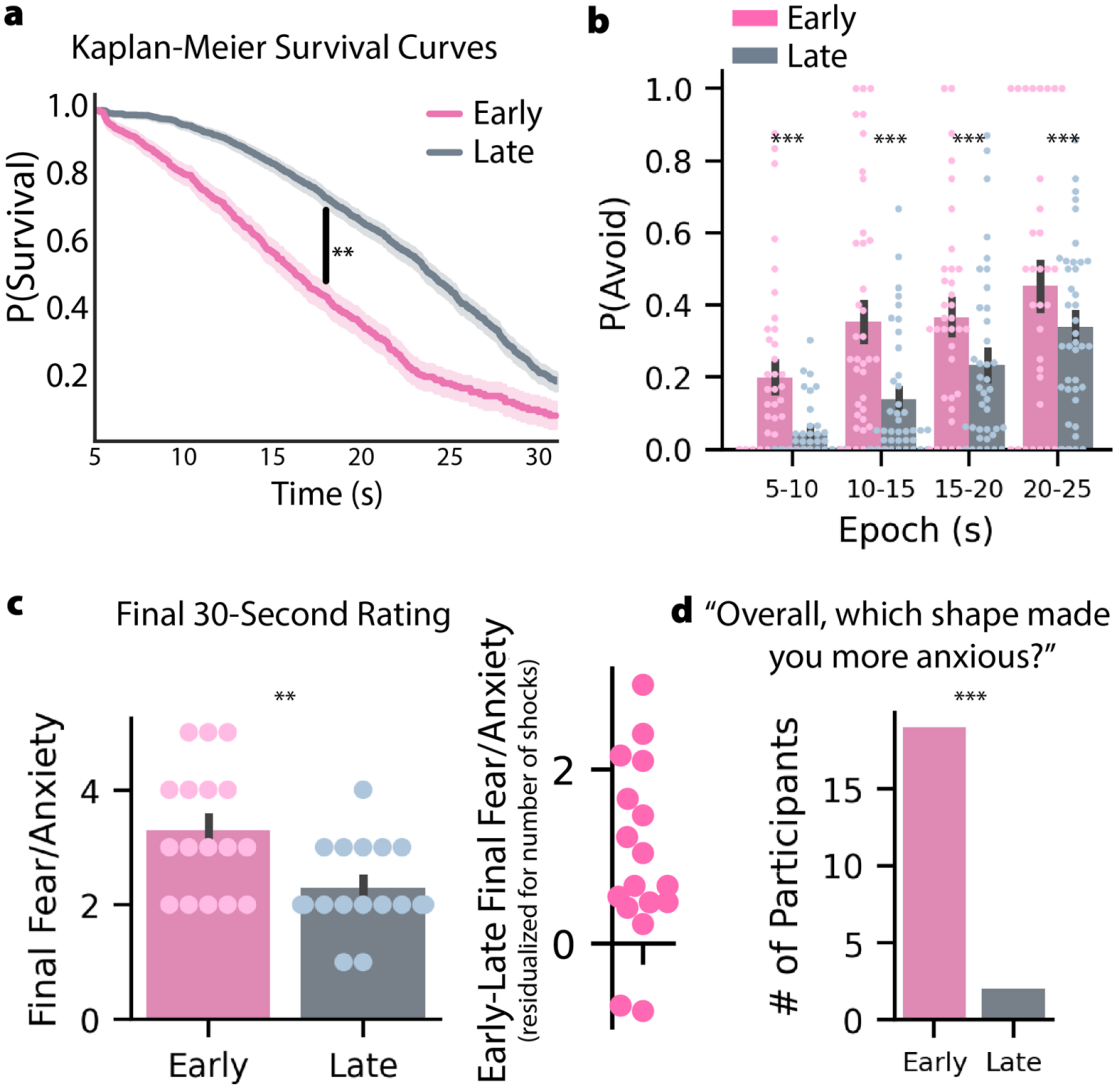
**Higher hazard rates cause more avoidance behavior and fear/anxiety. a)** Survival analysis revealed a significant difference between avoidance behavior at all timepoints (log-rank test, N = 42, 𝑥^2^ = 259.3, p < .005). **b)** In epochs during which P(Threat) values were matched (see Figure 1d), participants were significantly more likely to avoid the early-threat condition, where the hazard rate was higher (independent-samples *t*-tests, N = 42: [6–10s: *t* = 8.09, p < .001], [11–15s: *t* = 7.80, p < .001], [16–20s: *t* = 4.71, p < .001], [21–25s: *t* = 8.30, p < .001]). **c)** In a final, 30-second, unavoidable-shock trial for each condition (counterbalanced), participants reported significantly more fear/anxiety in the early-threat condition (Wilcoxon rank-sum test, N = 21, *U* = 328.5, p < .005). This analysis held after statistically accounting for the cumulative number of shocks received in each condition, as can be seen in the early-late fear/anxiety ratings residualized for the number of shocks (c, right). **d)** In a forced-choice test, 19 of 21 participants identified the early-threat condition as more anxiogenic (binomial test, N = 21, p < .001). Error bars represent the 68% CI, corresponding to the standard error of the mean. ** = p < .005, *** = p < .001.

Consistent with our hypothesis, participants behaved as though they were more likely to be shocked in the early-threat condition, even after the initial period of high P(Threat) had passed. Survival analysis of escape behavior modeling all trial outcomes (including those censored by shocks; see Supplemental Methods) demonstrated that participants were more likely to avoid the early-threat condition, forgoing cash rewards (log-rank test, N = 42, *χ*^2^ = 259.30, p < .005; Figure 2a). These data show that the hazard rate can drive avoidance behavior even when P(Threat) is matched between conditions (Figure 1d, top). In fact, participants were more likely to avoid the early-threat condition during all P(Threat)-matched epochs, including the 16–20s and 21–25s epochs in which they reported a *lower* likelihood of being shocked in the early-threat condition (Figures 1d, 2b, S2; independent-samples *t*-tests, N = 42: [6–10s: *t* = 8.09, p < .001], [11–15s: *t* = 7.80, p < .001], [16–20s: *t* = 4.71, p < .001], [21–25s: *t* = 8.30, p < .001]). This finding underscores the maladaptive potential of fear/anxiety, as participants’ behavior contradicted their reported understanding of the threat conditions. Ultimately, participants earned 17.8% less, on average, in the early-threat condition (paired-samples *t*-test = 9.29, p < .001).

### Higher hazard rates elicit greater self-reported fear/anxiety

To determine if hazard rate predicted fear/anxiety, subsets of participants rated their fear/anxiety in each condition at multiple timepoints: between learning and testing phases (*pre-testing*, N = 33), on a subset of testing-phase trials (*in-trial*, N = 39), and after a final 30-second unavoidable trial in each condition (*final trial*, N = 31). Details on assessments, analyses, and covariates can be found in Supplemental Methods. As predicted, there was a main effect of *condition* such that the early-threat condition was associated with increased fear/anxiety across pre-testing (MixedLM: z = 2.074, p < .05; Figure S3a), in-trial (MixedLM: z = 3.145, p < .005), and final trial assessments (ANCOVA: F = 4.84 p < .05; Figure 2c). In addition, we found hazard rate was significantly associated with increased self-reported fear/anxiety across pre-testing (MixedLM: z = 9.045, p < .001; Figure S3b) and in-trial assessments (MixedLM: z = 2.48, p < .05). Critically, these effects could not be accounted for by the number of shocks a participant received or the discrete probability of threat. Finally, in a forced-choice question posed at the end of the study—“*Overall, which shape made you more anxious*?”—19 of N = 21 participants chose the shape representing the early-threat condition (binomial test, p < .001; Figure 2d). Collectively, our findings compellingly demonstrate that the hazard rate is sufficient to drive changes in the emotional experience of fear/anxiety across uncertain contexts, even when discrete P(Threat) is held constant.

## Discussion

In uncertain environments with a higher hazard rate, participants were more likely to forgo rewards and experienced more fear/anxiety, indicating that individuals track the statistics of the environments to guide behavioral and emotional responses. Critically, we found that even when the hazard rate was at odds with the experimenter-determined or self-reported probability of shock, participants *behaved* as though the higher hazard rate condition was more threatening, demonstrating that the hazard rate is important for understanding avoidance behavior. Moreover, we found that variations in the hazard rate—which is necessarily higher during temporally uncertain vs. certain threat anticipation—were sufficient to alter fear/anxiety. Together, these results demonstrate that the hazard rate contributes to alterations in behavior and the experience of fear/anxiety. This likely reflects the accumulation of moments where an individual’s perception of threat probability is higher than the actual probability, due to their computation of hazard rate. Thus, increases in fear/anxiety in comparisons of uncertain-vs-certain threat anticipation (e.g. as seen in: Grillon et al., 2004; Grupe & Nitschke, 2013; Hur et al., 2020; Krain et al., 2008; Schmitz & Grillon, 2012; Simmons et al., 2008; Yassa et al., 2012), in part, reflect differences in the hazard rate, *not* uncertainty *per se*.

Our findings demonstrate that high-level concepts like “uncertainty” are reducible into dissociable component parts. This is the foundation of a statistical-learning approach to understanding fear/anxiety, which can also incorporate complementary, but categorically different aspects of uncertainty (e.g., Browning et al., 2015). Importantly, changes in the hazard rate impact any-and-all contexts in which the timing of a negative event is uncertain—even low-intensity or low-probability threats. Our statistical modeling approach gives evidence, for the first time, that hazard rate contributes to the experience of fear/anxiety. Although our study focused on dissociating hazard rates from discrete event probabilities, related statistical features may have contributed to our reported effects. Indeed, our observations could reflect a function of the hazard rate (e.g., cumulative hazard rate; see Supplemental Text). Our results set the stage for increasingly granular investigations into the temporal dynamics of real-time threat processing.

Our model echoes Hitchcock’s insight: Sustained anticipation of a negative event can lead to mounting anxiety as the hazard rate increases. Anxious psychopathology is often characterized by emotional distress in putatively safe contexts, leading to avoidance and missed opportunities. Our model suggests hazard rate estimates can disproportionately increase in response to imagined or exceptionally rare threats. This opens the door to identifying the precise mechanisms that lead to maladaptive avoidance and emotional distress characteristic of pathological anxiety by dissociating the probability of threat, hazard rate computations, and uncertainty *per se*. This computational re-imagining of uncertainty—a transdiagnostic marker of anxiety—provides a tractable framework for basic and clinical research aimed at understanding, preventing, and treating these conditions.

## Data Accessibility Statement

The codebase for our study’s shock-workup, stimulus-presentation, and shock-delivery software — as well as data collected — can be found here: https://github.com/foxlab-ucdavis/shock-study.

## Additional File

The additional file for this article can be found as follows:

10.5334/cpsy.105.s1 Supplementary Information. Supplemental Text, Supplemental Methods and Supplemental Figures.
