# Supplementary material for: Temporal Dynamics of Uncertainty Cause Anxiety and Avoidance
Source: Comput Psychiatr. 2024 Jun 20;8(1):85–91. doi: 10.5334/cpsy.105 (PMC11192096; doi:10.5334/cpsy.105)
Supplement: Supplementary Information. — Supplemental Text, Supplemental Methods and Supplemental Figures. [file cpsy-8-1-105-s1.pdf]

Supplemental Information

Temporal Dynamics of Uncertainty Cause Anxiety and Avoidance

Dan Holley[1,2], Erica A. Varga[3, 4], Erie D. Boorman[1,4], Andrew S. Fox[1,2]

Author Affiliations

[1] Department of Psychology, University of California, Davis, Davis CA, 95616, USA

[2] California National Primate Research Center, University of California, Davis, Davis CA, 95616, USA

[3] Center for Neuroscience, University of California, Davis, Davis CA, 95618, USA

[4] Center for Mind and Brain, University of California, Davis, Davis CA, 95616, USA

Supplemental Text

A note on "Fear" and "Anxiety"

A number of researchers have put forth a theory that distinguishes between fear and anxiety (Davis et al., 2010 ;Fanselow & Lester, 1988), in which fear refers to the response to phasic threats and anxiety refers to the response to sustained threats. We remain cautious about wholesale adoption of this language, as we and others have discussed in previous reviews (Daniel-Watanabe & Fletcher;Shackman & Fox, 2016). In short, it is unclear if the distinction between the terms 'fear' and 'anxiety' adequately capture the complexity of subjective experience in response to threat, nor does this reconceptualization of phasic vs. sustained threat match the dictionary definitions of "fear" and "anxiety" (Grogans et al., 2023). Moreover, a lack of consensus definitions for phrases like "phasic"/"sustained" or "certain"/"uncertain" can lead different studies to reach different conclusions. As such, we maintain that development of a precise nomenclature for the varied experiences that fall into the overarching category of fear and anxiety will require further research. Here, we refrain from distinguishing between these linguistic constructs in our manuscript, instead preferring to use the term 'fear/anxiety' or 'fear and anxiety'. Instead of targeting specific emotional experiences (i.e. fear vs. anxiety), we focus on manipulating previously unmodeled statistics of the environment to understand their contribution to threat-related experiences.

Implications for understanding the neurobiology of fear and anxiety.

Alongside the development of theories of phasic vs. sustained threats, researchers have been searching for distinct neural substrates underlying 'fear' and 'anxiety'. This has led to the emergence of theories suggesting that responses to phasic threats require the amygdala, whereas responses to sustained threats require the bed nucleus of the stria terminalis (BST). Evidence for this distinction includes neuroscience studies examining temporally certain vs. temporally uncertain threat anticipation (Davis et al., 2010;Walker et al., 2003;Somerville et al., 2013). These data have been taken as suggestive that temporally certain and uncertain threats are processed through partially distinct circuits in the brain (Davis et al., 2010;Avery et al., 2016). However, other studies have failed to replicate these findings and/or presented evidence

that the hypothesized distinction between amygdala and BST is inconsistent (Shackman & Fox, 2016; Gungor & Pare, 2016; Hur et al., 2020). Our observations that temporally certain and uncertain threats differ in the hazard rate suggest an alternative explanation, which we further examined by demonstrating that differences in hazard rate are sufficient to alter behavior and self-reported fear/anxiety. These data open the door to new theories and experiments that incorporate the statistics of the environment to better understand the distinct contributions of the amygdala and BST to threat processing. We posit that a refined understanding of these circuits can contribute to novel approaches for treating and preventing the suffering associated with anxiety and depressive disorders (Grogans et al., 2023).

Understanding of the statistical parameters that contribute to fear/anxiety.

Although the data described here provide compelling evidence that alterations in the hazard rate are sufficient to cause anxiety, this is only the tip of the iceberg. It remains unclear if feelings of fear/anxiety reflect changes in hazard rate *per se*, or if they more accurately reflect a function of the hazard rate (e.g. cumulative hazard). This relates a larger theoretical question about the relationship between feelings and moment-to-moment expected value (i.e. EV=pV, where the EV is expected value, p is probability, and V is value). More precisely, it remains unknown if the experience of fear and anxiety reflects the moment-to-moment expected value, if these experiences reflect an accumulation of the expected value over time, or if they are best captured by other functions of expected value (e.g. peak-end rule). We expect that hazard rate on its own is insufficient to explain our results, as the hazard rate on the final shock trial is equivalent in the brief moments preceding and following the shock. That said, we do not see this as clear evidence that the cumulative hazard rate is sufficient either, as we anticipate that, even in this simple task, there are other parameters that could contribute to increased feelings of fear/anxiety. Fully capturing the relationship between the statistics of the environment, behavior, and affect will require consideration of additional statistical features, such as anticipation (e.g. experienced-threat = EV_t[threat] + λ^1*EV_{t+1}[threat] + λ^2*EV_{t+2}[threat] + … λ^n*EV_{t+k}[threat], where EV = probability[threat]*value[threat], where EV_t is the expected value at time t, and λ is a discount term for future events). In short, these data provide critical evidence to reconceptualize previous studies focused on temporally certain and uncertain threats, and

are an impetus for additional research incorporating the statistics of the environment in understanding the manifestation of fear and anxiety.

Supplemental Methods

Threat-Probability Dynamics of Time-to-Event Analyses

Here, we investigate the impact of the evolving probability of an outcome during a period of anticipation. These values—i.e., the hazard rate, [h]—can be computed for any point in the anticipation period as:

$$h(t) = \frac{p(t)}{1 - P(t)}$$

Where, h(t) is the hazard rate, p(t) is the probability of the event occurring at this specific moment in time, and P(t) is the probability that the event will occur in a given time interval, i.e. up to time t. This cumulative probability, P(t), is defined as the sum of the perceived probability, p(t) from time 0 to t:

$$P(t) = \sum_{i=0}^{t} p(i)$$

During anticipation of an event where the exact time of the outcome is known, P(t) will equal zero until the delivery of the outcome. During anticipation of an event where the distribution of the negative event is uncertain, P(t) will increase over the interval in which p(t) is greater than 0. Thus, the denominator of h(t) decreases over time, resulting in the same probability [p(t)] being associated with increased hazard rate [h(t)] until the anticipation period ends. The cumulative hazard rate throughout a time interval is:

$$H(t) = \sum_{i=0}^{t} h(i)$$

Notably, the H(t) is unconstrained, meaning that it could increase without being bound by some maximum value. This means that, unlike the actual probability [p(t)] or the cumulative probability [P], the cumulative hazard rate [H] can exceed 100% by the time that an outcome occurs (i.e., t=T). We hypothesized that participants would use the hazard rate, as opposed to the experimenter-defined momentary probability of threat, or P(shock), to guide behavior, and that environments with a higher hazard rate would elicit more fear/anxiety than those with a lower hazard rate.

Participants

A total of 44 adults participated in our study. All volunteers were recruited via UC Davis' SONA paid research participation system. Our inclusion criteria were age (18-40), vision (20-20 or corrected to 20-20 with no color deficiency), and English language fluency. Our exclusion criteria were ongoing psychiatric treatment, history of psychiatric disorder or neurological disorder/injury, ongoing illicit drug use, and pregnancy or possibility of pregnancy. All activities were approved by, and conducted in strict accordance with, the policies of the UC Davis Institutional Review Board under authorization 1716796-1. Because our study featured aversive electrical shocks, in addition to informed consent all participants were repeatedly reminded that they could withdraw at any time. Two participants were dropped from our study because their shock tolerance exceeded our paradigm's maximum shock level, bringing our total cohort to N=42 (31F/11M, mean age=21.68 years, SD=3.12 years).

Data-Collection Timeline

The following timeline describes the chronology of data collection for participants who completed the full study. Detailed methods are described in subsequent Supplemental Methods sections.

1. *Consent*: each participant completes the informed consent process, study orientation, and pre-study knowledge check to ensure an understanding of study procedures.
2. *Shock workups*: each participant completes shock workups to establish individualized shock level.
3. *Learning phase*: each participant experiences 100 trials total and receives 50 pseudo-randomized unavoidable shocks in each condition while responding to periodic within-

trials questions to provide fear/anxiety self-reports (i.e., "How fearful/anxious did the last shape make you?").

4. *Pre-testing*: each participant estimates the timing of shocks in each condition as well as their level of fear/anxiety of experienced during each epoch in each conditions.
5. *Optional break*: each participant is offered the opportunity to use the restroom, stretch, and/or have a drink of water.
6. *Testing phase*: each participant experiences 100 trials total (50 for each condition) and makes avoid/persist decisions during each trial; fear/anxiety self-reports are again collected exactly as they were in 3).
7. *Post-testing*: each participant completes a final 30-second "how fearful/anxious?" rating with inescapable shock in each condition as well as a forced-choice assessment of which conditioned stimulus (i.e., early- or late-threat environment cue) elicited more anxiety (i.e., "Overall, which shape made you more anxious?”).

Shock Equipment

To administer shock stimuli, we used the STMEPM-MRI System (Biopac), which consists of a constant-voltage Stimulator Module (STM100C; range: ±10 V), Stimulus Isolation Adapter (STMISOC), Isolated Power Supply (IPS100C), and MRI-compatible Filter/Cable Set (MECMRI-STMISO). The Stimulator Module provides safe, fully programmable, real-time computer control over the electrical stimulus train (i.e., pulse duration, repetition, onset, and amplitude). The STMEPM-MRI System is intrinsically safe—despite possible errors in user stimulation setup or programming—under all operating conditions. Specifically, the strongest possible pulse under open-circuit conditions (160 mJ at 500 ohms, or 200 V) is well below the levels detailed in IEC 60601-2-10:2015 (max allowed: 300 mJ at 500 ohms, or 500 V), the harmonized, international regulatory standard relating to the safety of nerve and muscle stimulators. Stimuli were delivered to the musculature of the hand, between thumb and forefinger, via disposable electrodes. The duration of every shock administered during the study was 0.1s. Volunteers were fully informed of these aspects of shock delivery, without deception, during the informed consent process and again before data collection. During the informed consent process and prior to data collection, our team repeatedly stressed that participants could stop at any time, but no participants withdrew.

Shock Workups

We used a progressive workup method to determine an appropriate shock value for each participant. Participants were instructed to help us identify a shock level that was “uncomfortable but not unbearable” and were reminded that they “should prefer to not receive the shock.” To find this level, we began with a device output of 20 V and worked up as necessary in increments of 20 V toward a maximum of 200 V, administering two shocks at each intensity and obtaining positive verbal consent from the participant at each step before increasing the value. Once we arrived at the appropriate shock level, confirmed by each participant, that value was used for the

remainder of our study. Participants were informed that their shock value could not be changed once the experiment began, but that they were free to withdraw at any time for any reason (e.g., if the shocks became too intense or, conversely, if they were insufficient to motivate engagement in the task.)

Detailed Description of the Paradigm

Our paradigm consists of a learning phase and testing phase. The learning phase lasts roughly 30 minutes and is intended to expose participants to the temporal threat statistics of two "environments"—one in which shocks tend to occur early ($P[shock]_{EARLY}$={5s:.35, 10s:.13, 15s:.13, 20s:.13, 25s:.13, 30s:.13}), and another in which the shocks tend to occur late ($P[shock]_{LATE}$={5s:.13, 10s:.13, 15s:.13, 20s:.13, 25s:.13, 30s:.35}), as shown in Figure 1c, d. Each environment was represented by one of two distinct shapes, which were randomly assigned for each participant (Figure 1e). During learning, participants sat in front of a computer screen and were connected to the shock equipment. Watches, mobile phones, and other distracting and/or timekeeping devices were silenced, collected, and secured. Participants saw 50 presentations each of the early and late shapes in an order that was predetermined by randomly-selected trial structures drawn from six available structures (see *Block Generation*). Each shape appeared on screen following a 1-second intertrial interval (ITI, indicated by a + symbol in our figures), remained on screen for 5 to 30 seconds, and then disappeared with the administration of a shock, followed by the next ITI (Figure 1e). All stimulus feedback was administered at 5-second intervals. During learning, participants had no volitional control over whether they would be shocked (i.e., they could not avoid the shocks); this was visually represented by a lock icon in the upper-left corner of the screen. Importantly, participants were not given any specific information about the timing distributions of shocks or how those distributions related to the shapes, although they were instructed to pay close attention and told that "depending on the shape, the timing of the shocks might be different." We measured attention to infer learning by asking a one-back memory question—i.e., "Which shape did you just see?"—after every third shape. Participants were informed that failing to answer at least 75% of these questions correctly would result in withdrawal from the study; no participants were withdrawn under this criterion. On a subset of 25 trials (counterbalanced), participants self-reported their fear/anxiety post-trial by answering the computer-automated prompt, "How fearful/anxious did the last shape make you?"; ratings were collected on a 1-5 ordinal scale, with 1 representing "Not at all" and 5 representing "extremely." These in-trial learning data were not analyzed, as the results were not relevant to our hypotheses. After the learning phase, participants self-reported their memory of the average shock timing of each of the shapes and the number of shocks delivered by each shape during each of the six 5-second bins. Participants also rated each epoch on the aforementioned 1-5 scale by answering the following prompt: "You saw the circle [author's note: or "square"; counterbalanced] 50 times. Each time, the circle stayed on-screen for a while, then shocked you and disappeared. Please rate how fearful/anxious the circle made you at each of these time points. [author's note: each epoch is then listed and rated]" Following an optional break, the participants completed the testing phase.

During the testing phase, participants once again saw 50 presentations of each of the early and late shapes, with presentation order and shock timing once again predetermined by a second trial structure, randomly drawn from the five available structures that were not chosen for the learning phase. In the testing phase, participants earned 1¢ per second while shapes were on screen. If a trial continued beyond 5 seconds (i.e., if a shock had not yet been delivered), the aforementioned lock icon in the upper left corner of the screen would disappear, and pressing the computer's spacebar would allow participants to avoid shock administration, ending the trial and advancing them to the next trial. This was fully explained to participants, who were told, “If you are not shocked within the first 5 seconds, the lock icon will disappear, and then you can decide whether and when to avoid the shock by pressing the spacebar.” Participants were told that they would still earn rewards even if they received a shock on a given trial and were instructed to use what they learned about the shapes during learning to strike whatever balance between risks and rewards they preferred. Participants were again asked to perform a one-back attention task after every third trial. All participants scored above our threshold 75% accuracy, and it was rare for participants to be incorrect. Participants again self-reported their fear/anxiety on a subset of 25 trials, using the same procedure described in the previous paragraph.

In the post-testing phase, a cross-section of N=21 participants performed a final, unavoidable-shock trial for each of the two shapes. For these trials, participants were informed that they could not avoid the shock. Each shape (counterbalanced) was presented for the maximum time (30s) and terminated with the delivery of an unavoidable shock. Participants rated their fear/anxiety from 1 (none) to 5 (extreme) immediately following each shock. These same participants (N=21) also answered a two-option, forced-choice question, “Overall, which shape made you more anxious?” (counterbalanced).

Block Generation and Assignment

To create blocks of trials that recapitulated the statistical dynamics of our model, we first built a “perfect” exemplar block in which the probabilities for early and late threat stimuli adhered exactly to our model at all timepoints. As an unbiased approach to adding random noise to our environments, we generated 10,000 block structures consisting of 50 early and 50 late stimuli with timing randomly generated but bounded by the discrete probabilities of our exemplar’s bins (Figure 1d, top). We then narrowed these 10,000 randomly generated blocks down to 21 candidate blocks where the observed probabilities were at least 99% correlated to the overall statistics of our exemplar. Since each subject would experience two block schedules, we incorporated a process to ensure diversity between the order of stimuli in the blocks. To do this, we repeatedly computed a pairwise correlation matrix of the stimuli order for the candidate blocks, beginning with all 21 candidates and systematically eliminating the most highly correlated candidates until 6 “most dissimilar” candidates remained. All simulations and analyses were conducted in Python 3.8.3 using the pandas and NumPy libraries. Block assignment was carried out on a per-subject basis by selecting two blocks using numpy’s random.choice function (without replacement).

Statistical Analyses

All statistical analyses were performed in Python v3.8.3. Kaplan-Meier survival analyses were performed using Python’s lifelines library (Davidson-Pilon, 2023) v0.27.1, and differences between the curves were evaluated via a log-rank test using lifelines’ statistics.logrank_test function. Because we do not know how a subject would have behaved after they were shocked, shock events were censored to not bias the survival analyses. Independent samples t-tests and ANCOVAs were performed using pingouin v0.3.4. General linear models were fit using statsmodels v0.13.2. Mixed effects models were fit with subject as a random factor using the "lbfgs" method. For all tests, the predicted effects are reported. Linear models and t-tests were performed as follows:

Post-Learning Perceived Probability of Shock:
ttest(expected_number_shocks_in_epoch$_{[early_threat]}$,
expected_number_shocks_in_epoch$_{[late_threat]}$)

ttest('About how many seconds did it take for the <Early Cue> to shock you, on average?', 'About how many seconds did it take for the <Late Cue> to shock you, on average?')

Pre-testing Fear/Anxiety Ratings (Figure S3a, b):
```
smf.mixedlm("rating ~  threat_env ", df, groups=df['subj'] )
smf.mixedlm("rating ~  hazard ", df, groups=df['subj'] )
```

During Testing Fear/Anxiety Ratings (Figure S3c, d):
```
smf.mixedlm("rating ~ threat_env + prob + trial_timer +num_shocks_so_far", df, groups=df['id'] )
smf.mixedlm("rating ~ hazard + prob + trial_timer +num_shocks_so_far", df, groups=df['id'] )
```

During Testing Fear/Anxiety Rating Residuals (Figure S3d):
```
smf.mixedlm("rating ~  prob + trial_timer +num_shocks_so_far", df, groups=df['id'] )
```

Final Trial Fear/Anxiety Ratings (Figure 2c):
```
ancova(data=df, dv='final_rating', covar='num_shocks_so_far', between='threat_env')
```

Final Trial Fear/Anxiety Rating Residuals (Figure 2c, right):
```
smf.ols("final_rating ~ num_shocks_so_far", data=df )
```

Supplemental References

A Functional Behavioristic Approach to Aversively Motivated Behavior: Predatory Imminence as a Determinant of the Topography of Defensive Behavior. (1988). In *Evolution and Learning* (pp. 185–212). Lawrence Erlbaum Associates, Inc.

Avery, S. N., Clauss, J. A., & Blackford, J. U. (2016). The Human BNST: Functional Role in Anxiety and Addiction. *Neuropsychopharmacology*, *41*(1), 126–141. https://doi.org/10.1038/npp.2015.185

Daniel-Watanabe, L., & Fletcher, P. C. (2022). Are Fear and Anxiety Truly Distinct? *Biological Psychiatry Global Open Science*, *2*(4), 341–349. https://doi.org/10.1016/j.bpsgos.2021.09.006

Davidson-Pilon, C. (2019). lifelines: Survival analysis in Python. *Journal of Open Source Software*, *4*(40), 1317. https://doi.org/10.21105/joss.01317

Davis, M., Walker, D. L., Miles, L., & Grillon, C. (2010a). Phasic vs Sustained Fear in Rats and Humans: Role of the Extended Amygdala in Fear vs Anxiety. *Neuropsychopharmacology*, *35*(1), 105–135. https://doi.org/10.1038/npp.2009.109

Davis, M., Walker, D. L., Miles, L., & Grillon, C. (2010b). Phasic vs Sustained Fear in Rats and Humans: Role of the Extended Amygdala in Fear vs Anxiety. *Neuropsychopharmacology*, *35*(1), 105–135. https://doi.org/10.1038/npp.2009.109

Grogans, S. E., Bliss-Moreau, E., Buss, K. A., Clark, L. A., Fox, A. S., Keltner, D., Cowen, A. S., Kim, J. J., Kragel, P. A., MacLeod, C., Mobbs, D., Naragon-Gainey, K., Fullana, M. A., & Shackman, A. J. (2023). The nature and neurobiology of fear and anxiety: State of the science and opportunities for accelerating discovery. *Neuroscience & Biobehavioral Reviews*, *151*, 105237. https://doi.org/10.1016/j.neubiorev.2023.105237

Gungor, N. Z., & Pare, D. (2016). Functional Heterogeneity in the Bed Nucleus of the Stria Terminalis. *Journal of Neuroscience*, *36*(31), 8038–8049. https://doi.org/10.1523/JNEUROSCI.0856-16.2016

Hur, J., Smith, J. F., DeYoung, K. A., Anderson, A. S., Kuang, J., Kim, H. C., Tillman, R. M., Kuhn, M., Fox, A. S., & Shackman, A. J. (2020). Anxiety and the Neurobiology of Temporally Uncertain Threat Anticipation. *The Journal of Neuroscience*, *40*(41), 7949–7964. https://doi.org/10.1523/JNEUROSCI.0704-20.2020

Schmitz, A., & Grillon, C. (2012). Assessing fear and anxiety in humans using the threat of predictable and unpredictable aversive events (the NPU-threat test). *Nature Protocols*, *7*(3), 527–532. https://doi.org/10.1038/nprot.2012.001

Shackman, A. J., & Fox, A. S. (2016). Contributions of the Central Extended Amygdala to Fear and Anxiety. *Journal of Neuroscience*, *36*(31), 8050–8063. https://doi.org/10.1523/JNEUROSCI.0982-16.2016

Somerville, L. H., Wagner, D. D., Wig, G. S., Moran, J. M., Whalen, P. J., & Kelley, W. M. (2013). Interactions Between Transient and Sustained Neural Signals Support the Generation and Regulation of Anxious Emotion. *Cerebral Cortex*, *23*(1), 49–60. https://doi.org/10.1093/cercor/bhr373

Walker, D. L., Toufexis, D. J., & Davis, M. (2003). Role of the bed nucleus of the stria terminalis versus the amygdala in fear, stress, and anxiety. *European Journal of Pharmacology*, *463*(1–3), 199–216. https://doi.org/10.1016/S0014-2999(03)01282-2

Supplemental Figures

Supplemental Figure S1

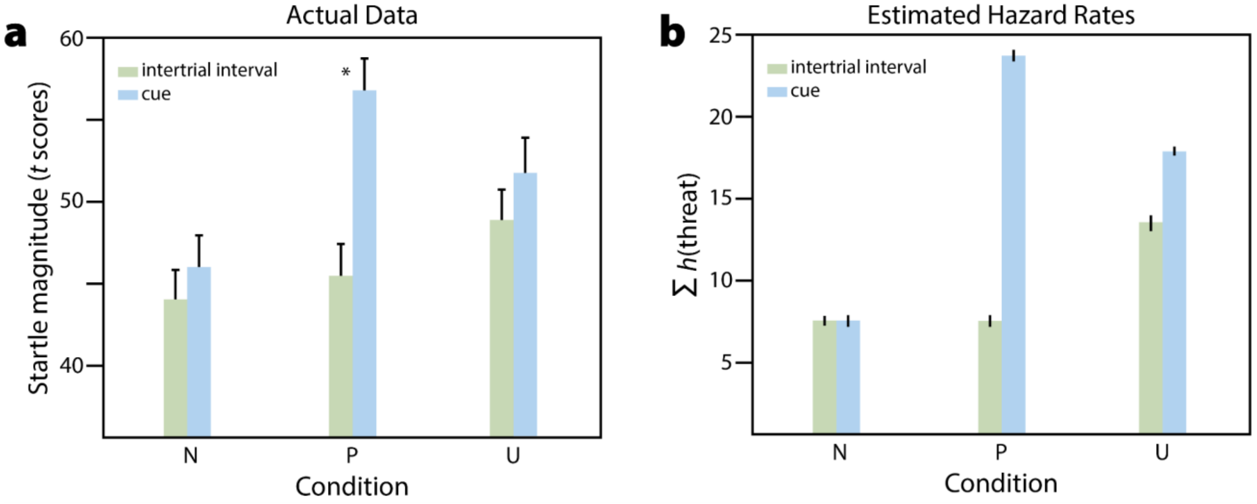

Figure S1. Previously published startle magnitude in the "*NPU-task*" (a) is similar to our estimate of the hazard rate based on their protocol (b) (Schmitz & Grillon, 2012). The x-axes reflect different conditions: Neutral (N), predictable (P), and Unpredictable (U). Hazard rate was computed using assuming full knowledge of the participant and are in arbitrary units.

Figure Supplemental Figure S2

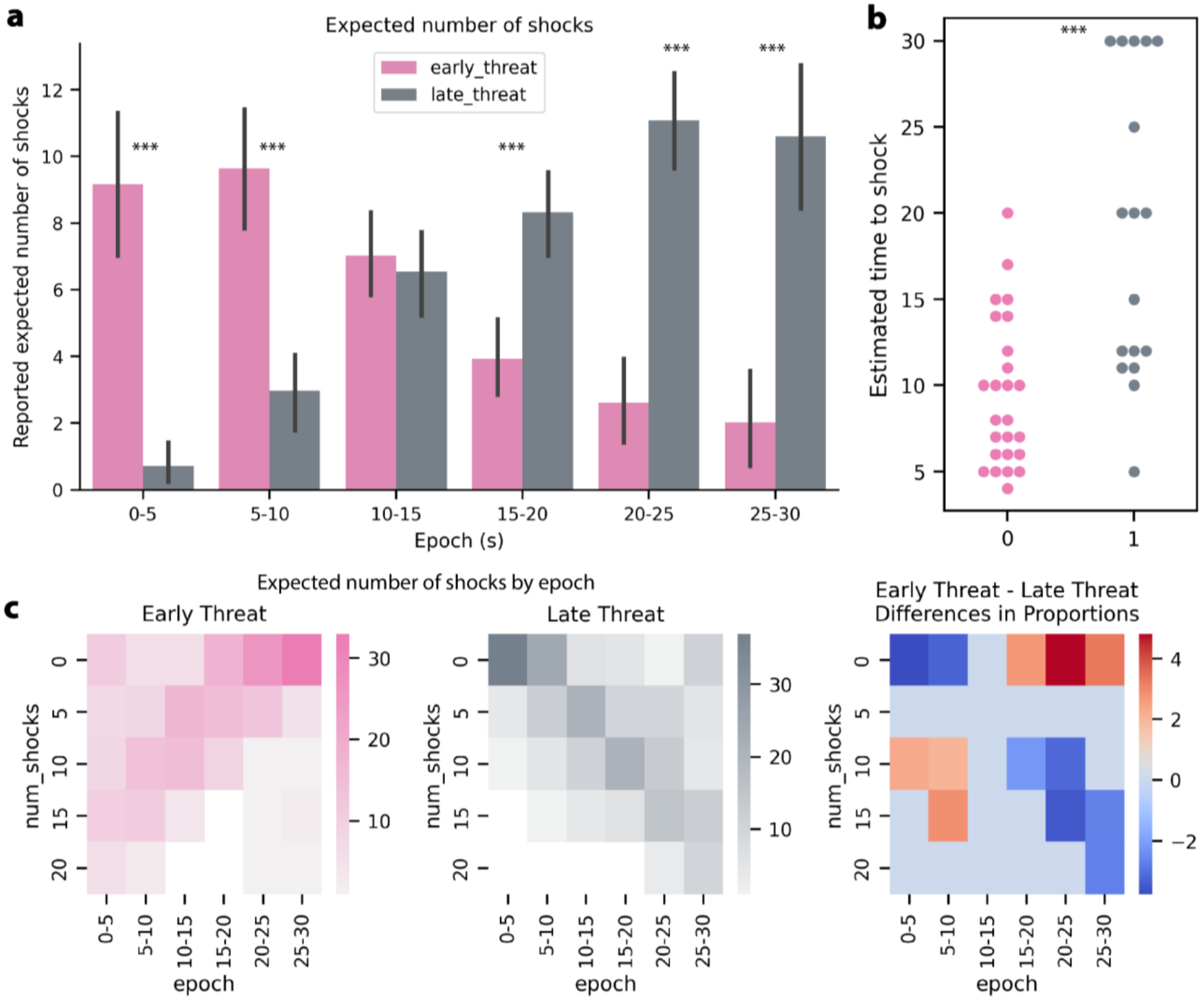

Figure S2. Participants reported perceiving shocks occurring earlier in the early-threat condition after the learning phase. In each condition, participants reported the estimated number of shocks occurring in each epoch (independent-samples *t*-tests, N=42: [0-5, t=7.36, p<.001], 5-10, t=6.15, p<.001], [10-15, t=0.51, p=0.60], [15-20, t=-4.87, p<.001], [20-25, t=-8.41, p<.001], [25-30, t=-6.09, p<.001]) (**a**) as well as the estimated average shock delivery time (**b**; t=4.17, p<0.001). Results demonstrated that participants expected more shocks in the early-threat condition during early epochs, and more shocks in the late-threat condition during late epochs. Replotting of estimated number of shocks by epoch reveals distinct patterns of expected shocks across the two conditions with significant differences in the predicted epoched shock trajectory (binomial test > .05) (**c**). Error bars indicate the 68% CI, corresponding to the standard error of the mean. *** = p<.001

Supplemental Figure S3

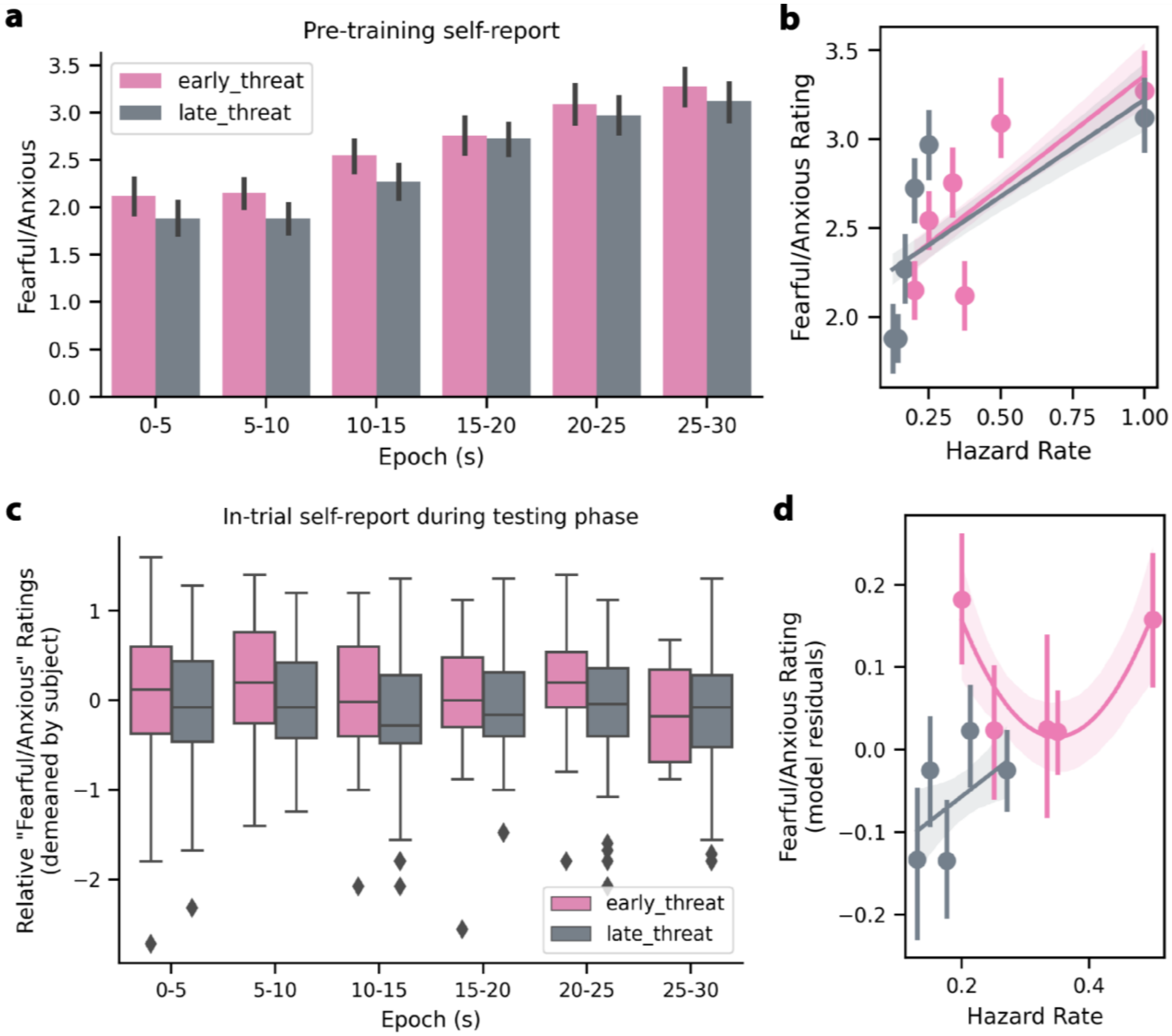

Figure S3. Retrospective reports of "Fearful/Anxious" experiences after learning (a) and during testing (c). After the learning phase, for each condition participants were asked between the learning and testing phases to report how "Fearful/Anxious" they were during each epoch (**a**) (MixedLM: z=2.074, p<.05): Self-reported ratings of fear/anxiety were significantly associated with hazard rate across conditions. (**b**) (MixedLM: z=9.045, p<.001): On a subset of learning trials in each condition participants were asked to report how "Fearful/Anxious" they were on that trial. The relative fear/anxiety ratings (demeaned by subject) are plotted by epoch. (**c**) (MixedLM: z=3.145, p<.005): To visualize the relationship between hazard-rate and self-reported ratings during testing (MixedLM: z=2.48, p<.05), we plotted the hazard rate against residual anxiety/fear-ratings after modeling other relevant factors (i.e., we fit [rating ~ prob + trial_timer + num_shocks_so_far", df, groups = ID] to get predicted_ratings without accounting for hazard rate, and model_residuals = actual_ratings - predicted_ratings). (**d**) (MixedLM: z=2.48, p<.05; see methods for details and main text for statistical analysis): Though obvious

effects are lacking, this likely reflects trial-by-trial biases during trials in which participants persisted, as well as variation and differences between the actual and perceived hazard rate (see Fig. S2). Error bars are the 68% CI, corresponding to the standard error of the mean.
